# Assessment of the Clinical Interactions of GAA Repeat Expansions in *FGF14* and *FXN*

**DOI:** 10.1212/NXG.0000000000200210

**Published:** 2024-11-20

**Authors:** Brandon J. Gerhart, David Pellerin, Matt C. Danzi, Stephan Zuchner, Bernard Brais, Gabriel Matos-Rodrigues, Andre Nussenzweig, Karen Usdin, Courtney C. Park, Jill S. Napierala, David R. Lynch, Marek Napierala

**Affiliations:** From the Department of Neurology (B.J.G., J.S.N., M.N.), O'Donnell Brain Institute, University of Texas Southwestern Medical Center, Dallas; Department of Neurology and Neurosurgery (D.P., B.B.), Montreal Neurological Hospital and Institute, McGill University, Montreal, Quebec, Canada; Department of Neuromuscular Diseases (D.P.), UCL Queen Square Institute of Neurology and The National Hospital for Neurology and Neurosurgery, University College London, United Kingdom; Dr. John T. Macdonald Foundation Department of Human Genetics and John P. Hussman Institute for Human Genomics (M.C.D., S.Z.), University of Miami Miller School of Medicine, FL; Department of Human Genetics (B.B.), McGill University, Montreal, Quebec, Canada; Laboratory of Genome Integrity (G.M.-R., A.N.), National Cancer Institute, NIH; Laboratory of Cell and Molecular Biology (K.U.), National Institute of Diabetes and Digestive and Kidney Diseases, National Institutes of Health, Bethesda, MD; and Department of Pediatrics and Neurology (C.C.P., D.R.L.), The Children's Hospital of Philadelphia, PA.

## Abstract

**Background and Objectives:**

The number of GAA repeats in the *FXN* gene is a major but not sole determinant of the clinical presentation of Friedreich ataxia (FRDA). The objective of this study was to establish whether the length of the GAA repeat tract in the *FGF14* gene, which is associated with another neurodegenerative disorder (SCA27B), affects the clinical presentation (age at onset, mFARS score) of patients with FRDA.

**Methods:**

The number of GAA repeats in the *FXN* and *FGF14* genes was determined using PCR in a cohort of 221 patients with FRDA. Next, we compared absolute lengths of the *FGF14* GAAs with *FXN* GAAs, followed by correlative analyses to determine potential effects of *FGF14* GAA length on age at onset and clinical presentation (mFARS) of FRDA.

**Results:**

We found no significant correlation between the size of the GAA repeats in *FXN* and *FGF14* loci in our FRDA cohort. Moreover, the number of GAAs in *FGF14* did not affect the clinical presentation of FRDA even in a small number of cases where a long *FGF14* allele was present.

**Discussion:**

Despite both molecular and clinical similarities between FRDA and SCA27B, the length of the GAA repeats in the *FGF14* gene, including potentially pathogenic alleles, did not influence the clinical presentation of FRDA.

## Introduction

Short polypurine-polypyrimidine tandem repeat tracts are frequently found in the human genome. One such repeat tract, a stretch of GAA trinucleotide repeats in intron 1 of the frataxin (*FXN*) gene, was identified almost 3 decades ago as the cause of Friedreich ataxia (FRDA).^1^ FRDA is an autosomal recessive neurodegenerative disorder caused by the biallelic expansion of these repeats, leading to transcriptional silencing of the mutated gene and reduced frataxin levels. While the pathogenic threshold starts at ∼60 GAA repeats, most patients carry alleles with 600–900 repeats, although alleles containing more than 1,000 repeats are frequently encountered.^2,3^ Nonpathogenic GAA alleles demonstrate a bimodal distribution, with short normal (SN) alleles (7–12 GAAs) found on 83% of chromosomes and long normal alleles (16–34 GAAs) found on 17% of chromosomes.^4^ Nonpathogenic alleles longer than 34 GAAs are very rare in the population.^4^ A strong correlation exists between length of the expanded *FXN* GAA repeats, especially the shorter (hereafter referred to as GAA1) allele, and age at onset and severity of many FRDA symptoms.^5-7^ However, the wide variation between GAA1 length and clinical presentation indicates the existence of other genetic and perhaps environmental modifiers of FRDA presentation.

Recently, expansion of a GAA repeat tract in intron 1 of the *FGF14* gene was shown to cause an autosomal dominant disorder, spinocerebellar ataxia 27B (SCA27B).^8,9^ Similar to FRDA, SCA27B is also a neurodegenerative disorder.^8,10-12^ A pathogenic threshold of at least 250 GAA repeats has been defined, although possibly with incomplete penetrance.^9,10^ Full penetrance is believed to occur above 300 or 335 GAA repeats.^9,10^ Recent work indicates that select features of SCA27B, such as downbeat nystagmus, are found more frequently in the population carrying 200–249 GAAs, suggesting that even shorter alleles may have pathogenic consequences.^13^ *FGF14* alleles in unaffected individuals demonstrate a large variation in repeat size, with neurologically healthy controls having anywhere from (GAA)_<25_ up to (GAA)_250-300_ repeats, but in rare cases, control alleles carried (GAA)_>300_ repeats.^14^

Given the high prevalence of *FGF14* alleles with large GAA repeats in the human population, we wondered whether such alleles might influence the age at onset and clinical presentation of FRDA. While the length of the GAA tract in *FGF14* may not reach the threshold required for pathology in otherwise unaffected individuals, it may exacerbate the effect of low frataxin levels in FRDA.

## Methods

### Amplification of FXN GAA Repeats

All FRDA genomic DNA samples were obtained from The Children's Hospital of Philadelphia. All patients provided written informed consent through CHOP IRB approved protocol 2609, the Friedreich Ataxia Clinical Outcome Measure Study. The concentration and purity of DNA were determined using a NanoDrop One spectrophotometer (Thermo Fisher Scientific). Amplification of GAA repeat expansions in the *FXN* gene was performed by long-range PCR using 2 sets of primers as described in Refs. 15,16. Primers used for amplification were:

FXN_SetI_F1: 5′-GGAGGGAACCGTCTGGGCAAAGG,

FXN_SetI_R1: 5′-CAATCCAGGACAGTCAGGGCTTT,

FXN_SetII_F2: 5′-GGCTTGAACTTCCCACACGTGTT,

FXN_SetII_R2: 5′-AGGACCATCATGGCCACACTT.

### Amplification of *FGF14* GAA Repeats

Amplification of GAAs in the *FGF14* gene was performed as described in Ref. 9 using primers:

FGF14_F1: 5′-AGCAATCGTCAGTCAGTGTAAGC,

FGF14_R1: 5′-CAGTTCCTGCCCACATAGAGC.

Reactions were performed in a 50 µL volume with 50 ng of genomic DNA using the FailSafe PCR System with mix D (cat. FS99250; Epicentre). To reduce heteroduplex formation, PCR reactions were cooled down from 94°C to 4°C at a rate of 1°C/min before agarose electrophoresis.

### Determination of GAA Repeat Number

The amplification products were resolved on 1.0–1.2% agarose gels stained with SYBR Safe DNA Gel Stain (Thermo Fisher Scientific). Lane analyses were performed using Image Lab 6.1 software (BioRad). The length of an expanded GAA tract was determined using the base pair size called by Image Lab 6.1, with the total number of GAA repeats calculated by subtracting the length of the sequences flanking the GAA repeats from the number of base pairs of the PCR product and dividing by 3. For *FXN* SetI: [Number of GAA repeats = (length of base pairs of a PCR product – 1,370)/3]; for *FXN* SetII: [Number of GAA repeats = (length of base pairs of a PCR product – 498)/3] – the size of each allele was then averaged over the 2 sets of primers to determine a patient's GAA repeat size for each allele; for *FGF14*: [Number of GAA repeats = (length of base pairs of a PCR product – 165)/3].

### Repeat-Primed PCR

Patients who had large amplification products by PCR underwent bidirectional repeat-primed PCR (RP-PCR) targeting the 5′-end and the 3′-end of the locus to ascertain the presence of a GAA repeat expansion, as described previously.^17^ A total of 12 DNA samples with *FGF14* (GAA2)_>200_ were analyzed. RP-PCR products were analyzed on an ABI 3730xl DNA Analyzer with a 50-cm POP-7 capillary using the GeneScan 1200 LIZ Dye Size Standard following the manufacturer's instructions. Results were analyzed with the GeneMapper software version 6 using the built-in microsatellite default settings. The presence of characteristic saw-toothed products indicated the presence of a GAA repeat expansion at the *FGF14* repeat locus. Chromatograms were inspected for the presence of interruptions.

### Data Analysis

We evaluated the influence of *FGF14* GAA values on FRDA in 2 ways. In the first analysis, we compared the lengths of *FGF14* GAA repeats with GAA1 and GAA2 of *FXN* in a subcohort of patients of FRDA followed in the Friedreich Ataxia Clinical Outcome Measures Study (FACOMS) (n = 221 in this sub-cohort),^18,19^ 184 of whom had detailed clinical information available. In FACOMS, *FXN* GAA1 lengths are available from values determined by commercial genetic testing, which could then be correlated with values directly obtained by the methodology described above. In the second analysis, we assessed whether *FGF14* GAA values correlated with or altered clinical features of FRDA. Analysis was performed using STATA (College Station, TX); data were initially evaluated using summary statistics, followed by linear and nonlinear correlations and, where relevant, linear regression. The incremental effect of *FGF14* GAA values on FRDA disease status was assessed by determining if the addition of *FGF14* GAA values to multiple linear regression of FRDA disease status (AOO accounting for *FXN* GAA1, age, and sex) improved R^2^ values.^20^ Twelve individuals carrying *FXN* point mutations were excluded from GAA1 correlations.

### Standard Protocol Approvals, Registrations, and Patient Consents

All patients provided written informed consent through CHOP IRB approved protocol 2609, the Friedreich Ataxia Clinical Outcome Measure Study.

### Data Availability

Anonymized data not published within this article will be made available by request from any qualified investigator.

## Results

**Figure 1:**
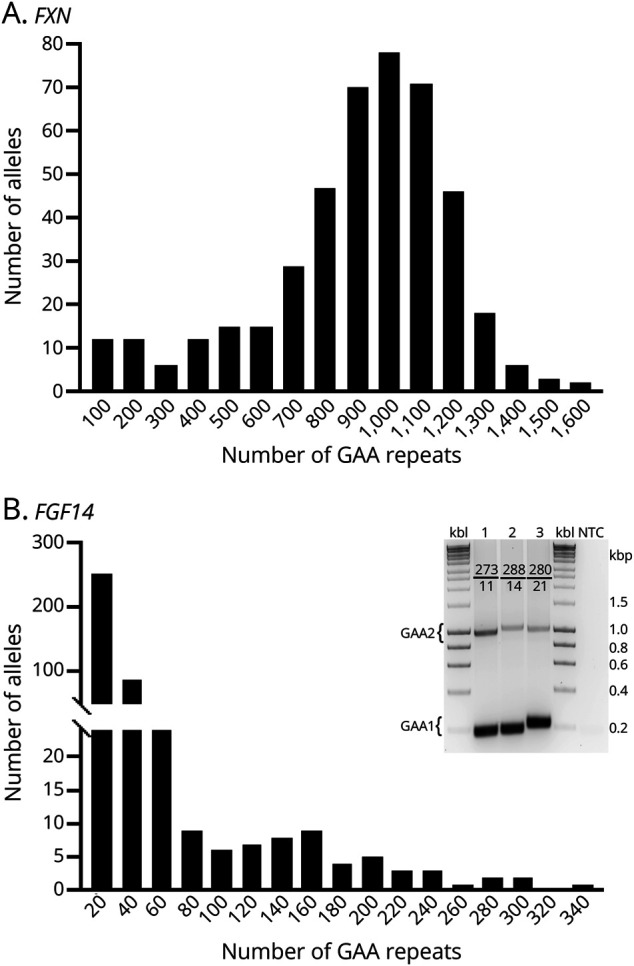
Lengths of *FXN* and *FGF14* GAA Repeats in the FRDA Cohort (A) Distribution *FXN* GAA Repeats in the FRDA cohort. A total of 442 alleles are plotted in 100 repeat bins. Twelve short alleles from compound heterozygous patients with FRDA are in the first bin (0–100 repeats. (B) Distribution of *FGF14* GAAs in the FRDA cohort. A total of 442 alleles are plotted in 20-repeat bins. Analysis of PCR amplification of long *FGF14* alleles in 3 samples is shown in the inset. Number of GAAs in *FGF14* GAA1 and GAA2 is indicated in each lane. FRDA = Friedreich ataxia; Kbl = 1 kbp DNA ladder; NTC = no template control.

The cohort of patients with FRDA was typical of the American FRDA population with a mean age at onset in late childhood/early adolescence (12.3 years), an equal distribution of men and women, a mean *FXN* GAA1 just slightly longer than the typical American mean, and a level of dysfunction (as measured by mFARS) of a symptomatic person not yet using a walking aid. A total of 221 samples (442 alleles) were analyzed for GAA repeat lengths in the *FXN* and *FGF14* genes. Of those, 209 patients with FRDA carried biallelic GAA expansions, whereas 12 were compound heterozygotes with a point mutation on one allele and a GAA expansion on the other allele. The number of *FXN* GAA repeats in patients with FRDA ranged from 112 to 1,591 (excluding short GAAs in compound heterozygous samples), where the average size of GAA1 and GAA2 was 727 GAAs and 1,026 GAAs, respectively (Figure 1A). Analysis of the *FGF14* gene in 221 FRDA patient samples using long-range PCR and agarose gel detection identified 12 patients with FRDA (5.43%) with (GAA)_>200_ [one patient with (GAA)_>300_, 5 with (GAA)_250-299_, and 6 with (GAA)_200-249_]. Validation was performed using long-range PCR and capillary electrophoresis, which found 3 alleles with (GAA)_>300_, 3 alleles with (GAA)_250-299_, and 6 alleles with (GAA)_200-249_ (eTable 1). Approximately 16% of alleles contained more than 50 GAA repeats (Figure 1B). Except for *FGF14* GAA1, data for each variable were normally distributed (Table 1). *FXN* GAA1 correlated with baseline age (*r* = −0.55), consistent with the fact that individuals carrying longer *FXN* GAA1 repeats present earlier with FRDA.

**Table 1:** Descriptive Statistics of the Friedreich Ataxia Subcohorts Assessed in This Study

Parameter	Mean	Median	Skewness	n
Baseline age (y)	24.5 + 15.3	19.9 (13.4–32.6)	1.01	184
mFARS	26.1 ± 10.2	25.7 (19.3–48)	1.60	183
Age at onset (y)	12.3 ± 8.1	11.0 (6.5–15)	1.83	184
Sex	88 men, 96 women			184
*FXN* GAA1 (Repeats)	727 ± 249	789 (589–916)	−0.74	209
*FXN* GAA2 (Repeats)	1,026 ± 182	1,025 (937–1,038)	−0.38	221
*FGF14* GAA1 (Repeats)	18 ± 14	14 (13–18)	5.2	221
*FGF14* GAA2 (Repeats)	58 ± 65	33 (17–60)	1.9	221

Next, we compared the different GAA lengths across patients (Table 2). There was a strong correlation of *FXN* GAA1 lengths with previously measured commercial *FXN* GAA1 values in FACOMS patients, thus validating the present methodology for GAA assessment. In addition, there was a small but significant negative correlation of *FXN* GAA1 with *FGF14* GAA1 (*r* = −0.15). In addition, there was a nonsignificant correlation of *FXN* GAA1 with *FGF14* GAA2 (*r* = −0.05). This suggests a slight tendency for *FGF14* GAA lengths to be shorter in patients with FRDA with long *FXN* GAA1 lengths.

**Table 2:** Correlations of Values Across Our Laboratory and Commercial Testing for *FXN* and *FGF14* GAA Repeats

Correlation	R2/*p* value	Regression coefficient
FXN GAA1 vs FACOMS FXN GAA1	0.61/<0.0001	0.78
FXN GAA1 vs FGF14 GAA1	0.022/0.027	−2.71
FXN GAA1 vs FGF14 GAA2	0.0029/0.43	−0.21

FACOMS *FXN* GAA1 was previously determined by commercial testing while *FXN* and *FGF14* GAA lengths were directly determined locally. n = 184.

We then examined the ability of GAA lengths to predict phenotypic severity in FRDA using AOO (Table 3) and mFARS scores (Table 4). As seen in previous studies, FRDA AOO was predicted by *FXN* GAA1 length (*p* < 0.0001), with longer *FXN* GAA1 values predicting earlier onset. When either *FGF14* GAA1 or *FGF14* GAA2 were added to the linear regression, there was no change in R^2^ value, and *FGF14* GAA values did not independently predict FRDA AOO. *FXN* GAA1 values remained highly significant, showing that only *FXN* GAA1 has any predictive value on FRDA AOO. Similarly, mFARS scores were predicted by age and *FXN* GAA1 values, but not sex; inclusion of *FGF14* GAA1 values had no effect on prediction of mFARS, showing that *FGF14* GAA1 values do not act as modifiers of FRDA phenotypic severity. Similar results were observed for *FGF14* GAA2.

**Table 3:** Linear Regression of FRDA AOO With GAA Lengths

Regression	R2(model only)/*p* value	Coefficient	n
AOO by *FXN* GAA1	0.49/<0.0001	−0.023 ± 002	184
Overall Model: AOO by *FXN* GAA1/*FGF14* GAA1	0.49/<0.0001		
*FXN* GAA1	0.000	−0.023 ± 0.002	
*FGF14* GAA1	0.25	0.055 ± 0.047	
Overall Model: AOO by *FXN* GAA1/*FGF14* GAA2	0.49/0.000		184
*FXN* GAA1	0.000	−0.023 ± 0 0.002	
*FGF14* GAA2	0.904	0.00080 ± 0.0066	

Abbreviation: FRDA = Friedreich ataxia.

Linear regression analysis examining predictive values of *FGF14* GAA1 on AOO in FRDA. Overall model R^2^ and *p* values are shown along with individual variable *p* value and individual variable regression coefficients. *FXN* GAA1 predicted AOO with high significance. R^2^ values did not increase when *FGF14* GAA1 or GAA2 was added to the model, showing the lack of effect of *FGF14* GAA length on FRDA AOO.

**Table 4:** Linear Regression of mFARS Score With GAA Lengths

Model	R2 (model only)/*p* value	Coefficients	n
mFARS by Age/*FXN* GAA1/sex	Overall Model: 0.30/<0.0001 Age: <0.0001 *FXN* GAA1: <0.0001 Sex: 0.148	Age: 0.44 ± 0.05 GAA1: 0.018 ± 0.003 Sex: 1.85 ± 1.28	183
mFARS by Age/*FXN* GAA1/sex/*FGF14* GAA1	Model: 0.30/<0.0001 Age: <0.0001 *FXN* GAA1: <0.0001 Sex: 0.137 FGF GAA1: 0.456	Age: 0.44 ± 0.05 GAA1: 0.018 ± 0.003 Sex: 1.9 ± 1.3 FGF GAA1: −0.054 ± 0.072	183

Linear regression analysis examining the predictive value of *FGF14* GAA1 on AOO in FRDA. Overall model R^2^ and *p* values are shown along with individual variable *p* value and individual variable regression coefficients. *FXN* GAA1 predicted AOO with high significance. R^2^ values did not increase when *FGF14* GAA1 was added to the model, showing the lack of effect of *FGF14* GAA1 values on FRDA AOO. Similar results were noted with *FGF14* GAA2 (not shown).

**Figure 2:**
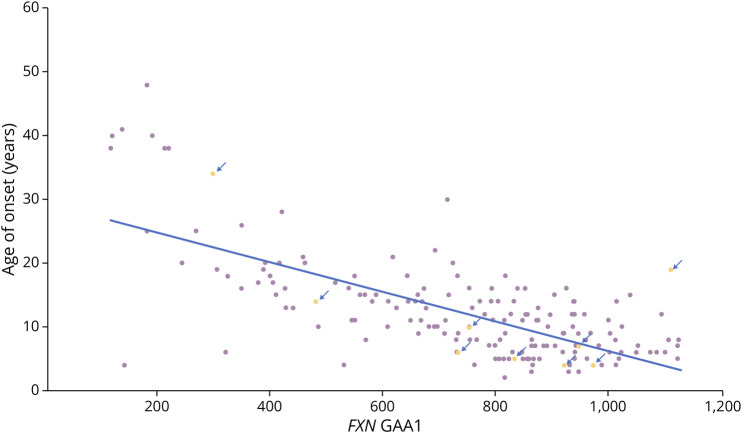
Effect of Long *FGF14* GAAs on AOO of FRDA Age at onset was plotted against *FXN* GAA1 repeat length for the entire FACOMS cohort (n = 174) and a best-fit line identified. Individuals with *FGF14* GAA2 values greater than 200 are identified by arrows. Such individuals are distributed above and below the best-fit line, showing graphically that *FGF14* GAA2 values do not influence AOO in FRDA. FRDA = Friedreich ataxia.

Six of the patients with FRDA carried long *FGF14* GAA2 alleles with more than 250 repeats that were within or near the disease range for SCA27B. Conceivably, this small group could have 2 forms of ataxia, leading to a more severe clinical course. To assess this, we plotted FRDA AOO vs *FXN* GAA1 allele length and determined the best-fit line. We then identified patients with *FGF14* GAA2_>200_ values who might have pathology arising from contributions from both the expansion at *FGF14* and expansions at *FXN*. On the graph, values from these individuals were split above and below the best-fit line, providing no evidence that a long *FGF14* GAA2 length accelerates the onset of FRDA (Figure 2).

Long *FGF14* alleles harboring non-GAA motifs [e.g., GAAGGA or (GAA)n(GCA)] are considered nonpathogenic.^8^ In addition, interruptions of the GAA tract affecting its purity may influence clinical presentation.^12^ Therefore, we used repeat-primed PCR (RP-PCR) to determine length and purity of the GAAs in the 12 patients with FRDA with GAA longer than 200 repeats (eTable 1). Of interest the 3 longest alleles were composed of non-GAA repeats. One allele with pure (GAA)_>250_ repeats was detected and 4 with GAA tracts ranging from 208 to 229 repeats. Finally, 4 alleles with 5′ end interruptions were identified harboring tracts of 203–249 of pure GAAs.

## Discussion

This study demonstrates the relative absence of physiologic interactions of GAA lengths in the *FXN* and *FGF14* genes. *FGF14* GAA lengths do not modify AOO or mFARS scores in FRDA, 2 indices of disease severity. In addition, correlations of *FXN* GAA1 with *FGF14* GAA lengths were low, providing little evidence for a mechanism that controls such lengths coordinately in cells. There was a slight but significant correlation of *FGF14* GAA1 length with *FXN* GAA1 length. The low magnitude suggests that the correlation results from a selection bias rather than a biological association. One explanation for this could be patient age. Samples from patients with long *FXN* GAA1 lengths present earlier, leading to a selection bias of long *FXN* GAA1 lengths being associated with younger patients (if samples are drawn early in the disease, as they usually are). If *FGF14* GAA1 lengths increase over time in cells, there would be small negative correlations between *FXN* GAA1 length and *FGF14* GAA lengths.

There seemed to be no interaction of the *FGF14* GAA repeats with FRDA clinical features. Not only did the length of *FGF14* GAA alleles not predict AOO or clinical severity (mFARS) in the overall cohort but individuals with long *FGF14* GAA2 alleles who might truly have 2 diagnoses do not seem to have an altered AOO in FRDA. This most likely reflects the fact that SCA27B is a slowly progressive, later onset disorder, making it difficult for a clinical interaction to be readily detectable in a typical cohort of patients with FRDA who are younger (several years before the typical onset of SCA27B). We cannot exclude the possibility that large *FGF14* GAA alleles have an effect on clinical presentation of other late-onset disorders. In addition, our conclusions are based on *FGF14*/*FXN* GAA repeat analyses in peripheral blood. However, symptoms of these ataxias are a consequence of pathologic changes predominantly in the nervous system (in FRDA also in cardiac and endocrine systems). It is possible that tissue-specific differences in somatic instability affecting the size of GAA repeats in both *FGF14* and *FXN* might influence the outcome of these analyses. Finally, analyses demonstrated that 7 out of 12 long GAA2 *FGF14* alleles were either interrupted or contained non-GAA repeats. Despite this fact, the remaining long alleles, including pure repeats of 262 and 249 GAAs, did not affect the clinical presentation of FRDA.

## Appendix Authors

**Table:**

Name	Location	Contribution
Brandon J. Gerhart, MS	Department of Neurology, O'Donnell Brain Institute, University of Texas Southwestern Medical Center, Dallas	Drafting/revision of the manuscript for content, including medical writing for content; major role in the acquisition of data; analysis or interpretation of data
David Pellerin, MD	Department of Neurology and Neurosurgery, Montreal Neurological Hospital and Institute, McGill University, Montreal, Quebec, Canada; Department of Neuromuscular Diseases, UCL Queen Square Institute of Neurology and The National Hospital for Neurology and Neurosurgery, University College London, United Kingdom	Drafting/revision of the manuscript for content, including medical writing for content; major role in the acquisition of data; analysis or interpretation of data
Matt C. Danzi, PhD	Dr. John T. Macdonald Foundation Department of Human Genetics and John P. Hussman Institute for Human Genomics, University of Miami Miller School of Medicine, FL	Drafting/revision of the manuscript for content, including medical writing for content; analysis or interpretation of data
Stephan Zuchner, MD, PhD	Dr. John T. Macdonald Foundation Department of Human Genetics and John P. Hussman Institute for Human Genomics, University of Miami Miller School of Medicine, FL	Drafting/revision of the manuscript for content, including medical writing for content; study concept or design; analysis or interpretation of data
Bernard Brais, MD, PhD	Department of Neurology and Neurosurgery, Montreal Neurological Hospital and Institute; Department of Human Genetics, McGill University, Montreal, Quebec, Canada	Drafting/revision of the manuscript for content, including medical writing for content; study concept or design
Gabriel Matos-Rodrigues, PhD	Laboratory of Genome Integrity, National Cancer Institute, NIH, Bethesda, MD	Drafting/revision of the manuscript for content, including medical writing for content; study concept or design; analysis or interpretation of data
Andre Nussenzweig, PhD	Laboratory of Genome Integrity, National Cancer Institute, NIH, Bethesda, MD	Drafting/revision of the manuscript for content, including medical writing for content; study concept or design; analysis or interpretation of data
Karen Usdin, PhD	Laboratory of Cell and Molecular Biology, National Institute of Diabetes and Digestive and Kidney Diseases, National Institutes of Health, Bethesda, MD	Drafting/revision of the manuscript for content, including medical writing for content; study concept or design
Courtney C. Park, BA	Department of Pediatrics and Neurology, The Children's Hospital of Philadelphia, PA	Drafting/revision of the manuscript for content, including medical writing for content; study concept or design; analysis or interpretation of data
Jill S. Napierala, PhD	Department of Neurology, O’Donnell Brain Institute, University of Texas Southwestern Medical Center, Dallas	Drafting/revision of the manuscript for content, including medical writing for content; study concept or design; analysis or interpretation of data
David R. Lynch, MD, PhD	Department of Pediatrics and Neurology, The Children's Hospital of Philadelphia, PA	Drafting/revision of the manuscript for content, including medical writing for content; study concept or design; analysis or interpretation of data
Marek Napierala, PhD	Department of Neurology, O’Donnell Brain Institute, University of Texas Southwestern Medical Center, Dallas	Drafting/revision of the manuscript for content, including medical writing for content; study concept or design; analysis or interpretation of data

## Glossary

FRDA: Friedreich ataxia
